# Morphological changes and their associations with clinical parameters in children with nephropathic cystinosis and chronic kidney disease prior to kidney replacement therapy over 25 years

**DOI:** 10.1007/s00467-024-06421-6

**Published:** 2024-06-08

**Authors:** Malina Brügelmann, Sophia Müller, Alina V. Bohlen, Katharina Hohenfellner, Anja Büscher, Markus J. Kemper, Kerstin Fröde, Nele Kanzelmeyer, Jun Oh, Heiko Billing, Jutta Gellermann, Dominik Müller, Lutz T. Weber, Birgit Acham-Roschitz, Klaus Arbeiter, Burkhard Tönshoff, Martina Hagenberg, Mislav S. Žebec, Dieter Haffner, Miroslav Zivicnjak

**Affiliations:** 1 Department of Pediatric Kidney, Liver and Metabolic Diseases, Hannover Medical School Children’s Hospital, Carl-Neuberg-Str. 1, 30625 Hannover, Germany; 2 Division of Pediatric Nephrology, Children’s Hospital, Rosenheim, Germany; 3 Department of Pediatrics II, University Hospital Essen, Essen, Germany; 4 Asklepios Hospital, Hamburg, Germany; 5 Department of Pediatric Nephrology, University Children’s Hospital Hamburg, Hamburg, Germany; 6 Clinic for Pediatric and Adolescent Medicine, RHK Clinic Ludwigsburg, Ludwigsburg, Germany; 7 Department of Pediatric Gastroenterology, Nephrology and Metabolic Diseases, Charité Universitätsmedizin Berlin, Berlin, Germany; 8 Pediatric Nephrology, Faculty of Medicine and University Hospital, Children’s and Adolescents’ Hospital, University of Cologne, Cologne, Germany; 9 Department of Pediatrics, Medical University Graz, Graz, Austria; 10 Division of Pediatric Nephrology and Gastroenterology, Medical University Vienna, Vienna, Austria; 11 Department of Pediatrics I, University Children’s Hospital Heidelberg, Heidelberg, Germany; 12 Children’s Hospital St. Elisabeth and St. Barbara, Halle (Saale), Germany; 13 Institute for Anthropological Research, Zagreb, Croatia

**Keywords:** Infantile nephropathic cystinosis, Chronic kidney disease, Body fat mass, Thoracic proportions, Growth, Body mass index

## Abstract

**Background:**

Infantile nephropathic cystinosis (INC) is a rare lysosomal storage disorder, mostly and often firstly affecting the kidneys, together with impaired disharmonious growth and rickets, eventually resulting in progressive chronic kidney disease (CKD). With the introduction of cysteamine therapy, most pediatric patients reach adulthood with no need for kidney replacement therapy. Still, detailed changes in INC patients’ clinical and morphological presentation over the past decades have not yet been thoroughly investigated.

**Methods:**

Two groups with a respective total of 64 children with INC and 302 children with CKD, both treated conservatively and aged 2 to 18 years, were prospectively observed in the time span from 1998 to 2022 with 1186 combined annual clinical and morphological examinations clustered into two measurement periods (1998 to 2015 and ≥ 2016).

**Results:**

In INC patients, thoracic proportion indices remained markedly increased, whereas body fat stores remained decreased over the past 25 years (+ 1 vs. below ± 0 *z*-score, respectively). Their CKD peers presented with overall improved growth, general harmonization of body proportions, and improved body fat stores, while INC patients only presented with an isolated significant increase in leg length over time (∆0.36 *z*-score). eGFR adjusted for age did not significantly change over the past 25 years in both groups. Alkaline phosphatase (ALP) showed a significant decrease in CKD patients over time, while remaining above normal levels in INC patients.

**Conclusions:**

Disproportionate thoracic shape and impaired body fat stores remain the most characteristic morphological traits in INC patients over the past 25 years, while causal mechanisms remain unclear.

**Graphical Abstract:**

**Figure:**
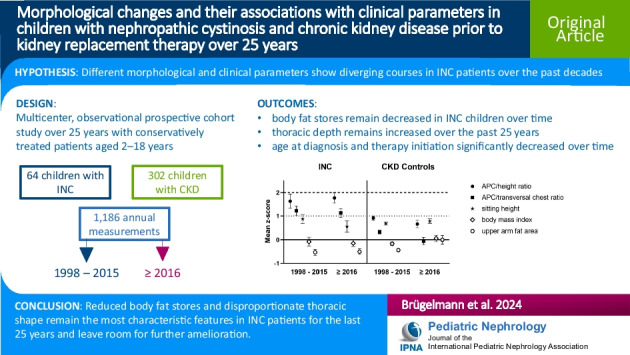
A higher resolution version of the Graphical abstract is available as Supplementary information

**Supplementary Information:**

The online version contains supplementary material available at 10.1007/s00467-024-06421-6.

## Introduction

Infantile nephropathic cystinosis (INC) is a rare lysosomal storage disorder with multisystem implications caused by intracellular accumulation of cystine crystals [1, 2]. The aggregation of crystals in internal organs was first described in 1903 by Kaufmann, which were later classified as cystine crystals by Abderhalden [3], and usually firstly results in generalized proximal tubule dysfunction (De Toni–Debré–Fanconi syndrome) thus causing symptoms such as polyuria and failure to thrive as well as electrolyte imbalances [1, 4, 5]. The disease typically manifests within the first 18 months of life and results in progressive chronic kidney disease (CKD) with numerous other complications such as malnutrition [6], hypophosphatemic rickets, myopathy [7], corneal deposits [8], and central nervous system complications [9], generally followed by kidney failure at around 10 years if not causally treated [10, 11]. Due to advances in dialysis therapy and graft surgery in the 1970s [12], progressive kidney failure did not necessarily result in death anymore but was alleviated by use of procedures replacing impaired kidney function. This was further enhanced in 1997 with the introduction of cystine-depleting therapy in Europe which prolonged the pre-terminal period**,** delayed the onset of complications, and increased life expectancy [13, 14]. To date, INC patients present with characteristic impairments in body growth and composition, i.e., disproportionate short stature with short legs and increased chest depth and reduced body fat [15, 16]. However, the dynamic changes in body morphology of INC children over the past decades remain largely unknown. To address these gaps, anthropometric parameters and their associations with clinical parameters were assessed in 64 children with INC and 302 CKD peers to facilitate a detailed insight into the disease and how its clinical and morphological appearance has shifted over the past 25 years.

## Material and methods

### Study design and patients

The prospective observational cohort study “CKD growth and development” was initiated in May 1998 at the Department of Pediatric Nephrology, Charité-Universitätsmedizin Berlin, Berlin, Germany, and expanded in the year 2000 to Hannover Medical School, Hannover, Germany, where in 2016 the complementing prospective multicenter cohort study “Growth and cognitive-motor abilities in children with nephropathic cystinosis and chronic kidney disease” commenced. CKD controls continued to be measured and examined at Hannover Medical School whereas patients with INC were also measured in nine additional German hospitals as well as two centers in Austria. For the analysis of treatment initiation and age at diagnosis, all children with INC (*N* = 88) were assessed. From this overall sample of INC and CKD patients with CKD stages 1–5 prior to kidney replacement therapy (KRT) [17], patients with acquired causes of CKD (e.g., hemolytic uremic syndrome) and/or complex multi systemic diseases (e.g., Bartter or Denys-Drash syndrome) with a possible secondary impact on growth and development were excluded. The decision to solely include conservatively treated children in this presented analysis is based on findings showing that INC patients treated early and adequately with cysteamine require KRT less frequently today, making conservative treatment of most research interest [13]. In total, 64 children with INC (32 male 32 female) and 302 with CKD (185 male 117 female) due to other congenital or hereditary kidney diseases (CKD controls) were eligible for this analysis. Patients were aged between 2 and 18 years and underwent 1186 annual measurements (INC, 193; CKD, 993) with an average of 3.24 annual measurements per patient (INC, 3.02; CKD, 3.29). INC and CKD patients’ measurements were divided into two measuring periods (clusters): the first ranging from May 1998 to December 2015 (1998–2015) and the second ranging from January 2016 to December 2022 (≥ 2016). These clusters were chosen to best depict changes between the two periods with varied medication and therapy options and to compare children treated and diagnosed early with those who were diagnosed much later. All INC patients within this study received cysteamine treatment. Clinical appointments included monitoring of biochemical parameters as well as individual medication, physical examinations, and anthropometric measurements. Approval by the ethics committee was obtained from the institutional review boards at each study site. The study was performed in accordance with the Declaration of Helsinki. All parents/guardians gave their written consent as well as patients where age appropriate.

### Methods

Standard laboratory techniques were used for determination of blood/serum levels of hemoglobin, albumin, creatinine, potassium, calcium, phosphate, alkaline phosphatase (ALP), and parathyroid hormone (PTH). Rates of anemia, hypokalemia, hypophosphatemia, and hypocalcemia were calculated using age- and sex-dependent reference intervals [17–19]. Serum calcium was corrected for serum albumin [20]. Metabolic acidosis was defined as a serum bicarbonate < 22 mmol/L [18, 19]. Estimated glomerular filtration rate (eGFR) was calculated based on the revised Schwartz equation [21]. Analysis of leukocyte cystine content levels was not feasible due to a lack of data from earlier time points. Annual anthropometric measurements included linear (height, sitting height, leg length) and transversal (chest depth and width) body dimensions and upper arm skin folds, while body mass index (BMI), sitting height index (ratio of sitting to total body height), upper arm fat area (UFA), and thoracic indices (chest depth to height ratio and chest depth to chest width ratio) were calculated as described in previous works [15, 16]. UFA and BMI were used as indirect markers of body composition. All measurements were performed by the same investigator (MZ) and taken in accordance with the International Biological Program using standardized equipment. *z*-scores regarding anthropometric parameters were calculated using reference data obtained from 5260 healthy children [22, 23]. All children received regular dietary counselling. Age at diagnosis and therapy initiation was collected from patients’ personal health records, while age at menarche was documented in female patients during examinations.

### Statistical analysis

This study expands upon a previously published study within this project which was initiated in 2016 [15, 16]. Descriptive statistics are given in terms of mean and standard deviation or estimated marginal mean (Est. MM) and 95% confidence interval (for repeated measurements), or median with interquartile range (IQR), or incidence percentage (%), as suitable for measurement scale/model and normality of distribution. Distribution normality was evaluated by using the Shapiro–Wilk test. To compare two groups of independent measurements either the Mann–Whitney test or *t*-test was used, as appropriate. To test differences between incidences of clinical and characteristics of medication in the two measurement clusters (1998–2015 vs. ≥ 2016) within the INC and CKD groups, chi-square test was used (MedCalc Software Ltd. version 22.014). Linear mixed-effect models (LMM) (repeated measurements with setting intercept and subject ID as random effect) were used to test differences in clinical/biochemical indicators between the two measurement clusters in INC and CKD patients with various numbers of measurements as well as varying time gaps amid measurements. Associations of anthropometric (leg length, anterior–posterior chest (APC) height ratio, and upper arm fat area) and clinical/biochemical characteristics (covariates: age, eGFR, serum bicarbonate, hemoglobin, sodium, potassium, calcium, phosphate, ALP, PTH) were also analyzed by use of LMM by setting the intercept and subject ID as random effect with unstructured covariance matrix type (UN). In the aforementioned LMM analysis, information criteria were used to choose the most appropriate model for each analysis and parameter group. Additionally, as mean age varied significantly between the two clusters, we analyzed biochemical parameters separately between the two clusters for INC and CKD patients by using LMM adjusted for age. Results were considered significant at a level of *p* < 0.05. SPSS for Windows, version 28.0 (IBM Corporation, NY, USA), was used. Graphs were generated by use of GraphPad Prism 9.3.1 (GraphPad Software, Inc., San Diego, CA).

## Results

### Patient characteristics and biochemical parameters

**Fig. 1:**
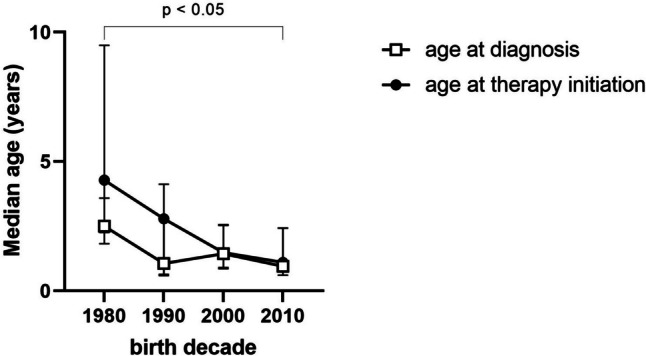
Ages at diagnosis of cystinosis and initiation of cysteamine therapy in 88 INC patients (entire sample regardless of later KRT status, see methods) presented by respective birth decade (decade 1980 = 01.01.1980–31.12.1989…) with interquartile range

Patient characteristics, as well as clinical and biochemical parameters, are presented in Tables 1 and 2. When analyzing the entire INC sample (all measurements from 1998 to 2022, regardless of KRT status, and with birth decades ranging from 1980 to 2010s), age at diagnosis (median 2.50 (IQR 1.8, 3.6) to 0.95 years (IQR 0.6, 2.4)) as well as age at initiation of cysteamine therapy (median 4.28 (IQR 2.3, 9.5) to 1.10 years (IQR 0.7, 2.4)) decreased significantly from birth decade 1980 to decade 2010 (both *p* < 0.05, Fig. 1). Narrowing in from this broader patient cohort, we further analyzed solely conservatively treated patients as described in the methods. Comparing the two measurement clusters (1998–2015 vs. ≥ 2016), patient age (at measurement) increased significantly in the INC group from 6.31 years to 11.10 years and in the CKD group from 8.76 years to 12.28 years (both *p* < 0.001, Table 1). Within each respective cluster, INC and CKD patients were comparable with regard to age distribution at measurement. With regard to distribution of sex, INC and CKD controls did not differ significantly in both measurement clusters. Congenital causes of CKD within the observed CKD patients were increased in the measurement cluster after 2015 (78.0% vs. 90.7%, *p* < 0.01). Raw eGFR declined significantly in both groups over time, but when adjusted for age, no significant change was observed (Table 1). Although administration of erythropoietin therapy decreased in both groups (only significantly in the CKD group), the incidence of anemia declined significantly in both groups over time (Table 2). A significant increase in hypokalemia rates in the INC group (34.1 to 50.0%, *p* < 0.05) was observed from first to second measurement cluster, whereas CKD patients presented with respectively generally lower and decreasing rates (9.8 to 3.4%, *p* < 0.01). ALP levels significantly declined in the CKD group from first to second measurement cluster. In the INC group, ALP *z*-scores remained elevated above normal across the observed time frame (Table 1). The administration of calcium and calcitriol therapy markedly decreased over time in patients with CKD. The administration of growth hormone treatment declined in both groups when compared to the earlier measurement cluster, but not significantly (INC 52.1 to 50.0%, CKD 30.6 to 27.5%, both *p* > 0.05, Table 2). In the period after 2015, both INC and CKD patients received vitamin D (primarily in the form of cholecalciferol) and phosphate therapy significantly more often than in the preceding measurement cluster (Table 2). From 1998 to 2015, all INC patients were treated with immediate release (IR) cysteamine at mean doses of 1179.10 mg/m^2^ body surface area (95% CI 1048.56–1309.62), of which two patients switched to delayed release (DR) cysteamine by 2015. In the subsequent measurement cluster, the majority of patients were switched to DR cysteamine or were started on DR cysteamine from the beginning, which was given at mean doses of 1074.38 mg/m^2^ body surface area (95% CI 925.80–1223.00).

**Table 1:** Biochemical parameters in conservatively treated INC and CKD patients analyzed by measurement cluster (1998–2015 vs. ≥ 2016)

	INC					CKD controls				
Time frame of measurements	1998–2015		≥ 2016			1998–2015		≥ 2016		
	Est. MM (95% CI)	N	Est. MM (95% CI)	*N*	*p*	Est. MM (95% CI)	*N*	Est. MM (95% CI)	*N*	*p*
Age, years	6.31 (4.98–7.65)	94 of 94	11.10 (9.91–12.28)	99 of 99	0.000	8.76 (8.20–9.33)	771 of 771	12.28 (11.57–12.98)	222 of 222	0.000
eGFR, mL/min per 1.73 m^2^	75.45 (63.90–87.01)	89 of 94	52.26 (41.64–62.88)	93 of 99	0.000	40.58 (37.14–44.01)	752 of 771	31.39 (27.60–35.18)	218 of 222	0.000
HCO_3_, mmol/L	22.36 (21.48–23.25)	87 of 94	22.89 (22.10–23.68)	81 of 99	0.321	23.11 (22.86–23.36)	718 of 771	22.72 (22.33–23.11)	210 of 222	0.064
Hemoglobin, g/dL	12.06 (11.47–12.66)	89 of 94	12.46 (11.95–12.97)	97 of 99	0.190	11.98 (11.80–12.16)	754 of 771	12.15 (11.90–12.40)	217 of 222	0.157
Sodium, mmol/L	139.45 (138.67–140.22)	89 of 94	139.67 (139.00–140.33)	97 of 99	0.644	139.97 (139.70–140.25)	739 of 771	140.37 (139.94–140.80)	217 of 222	0.100
Potassium, mmol/L	3.92 (3.78–4.07)	89 of 94	3.84 (3.71–3.96)	98 of 99	0.338	4.48 (4.42–4.54)	739 of 771	4.54 (4.46–4.63)	217 of 222	0.170
Calcium *z*-score	− 2.95 (− 4.08–1.81)	87 of 94	− 1.84 (− 2.78–0.89)	96 of 99	0.101	− 0.19 (− 0.47–0.09)	742 of 771	0.20 (− 0.25–0.65)	210 of 222	0.118
Phosphate *z*-score	− 0.42 (− 0.93–0.09)	85 of 94	− 0.01 (− 0.44–0.43)	88 of 99	0.160	1.04 (0.90–1.18)	729 of 771	0.85 (0.64–1.06)	207 of 222	0.096
ALP *z*-score	1.85 (1.23–2.47)	81 of 94	1.49 (0.97–2.01)	88 of 99	0.300	0.52 (0.31–0.73)	586 of 771	0.02 (− 0.32–0.35)	180 of 222	0.007
PTH *z*-score	2.20 (1.21–3.18)	80 of 94	2.01 (1.14–2.87)	86 of 99	0.710	3.17 (2.92–3.42)	614 of 771	2.97 (2.60–3.33)	201 of 222	0.283

Data is presented as *z*-scores and estimated marginal means with 95% confidence intervals. *p* values are based on pairwise comparisons (linear mixed models). *Est. MM* estimated marginal means, *eGFR* estimated glomerular filtration rate, *ALP* alkaline phosphatase, *PTH* parathyroid hormone

**Table 2:** General, clinical and therapy characteristics of conservatively treated pediatric INC and CKD patients

	INC			CKD controls		
Measurement clusters	1998–2015	≥ 2016		1998–2015	≥ 2016	
	% (*N* from total)	% (*N* from total)	*p*	% (*N* from total)	% (*N* from total)	*p*
Anemia	39.3% (35 of 89)	17.3% (17 of 98)	0.001	38.1% (287 of 754)	26.7% (58 of 217)	0.002
- EPO therapy	25.0% (20 of 80)	21.9% (21 of 96)	0.629	41.1% (232 of 564)	29.2% (57 of 195)	0.003
- Iron therapy	27.5% (22 of 80)	20.8% (20 of 96)	0.300	34.2% (193 of 564)	34.9% (68 of 195)	0.859
Hypokalemia	34.1% (28 of 82)	50.0% (47 of 94)	0.034	9.8% (67 of 686)	3.4% (7 of 207)	0.004
- Potassium therapy	72.5% (58 of 80)	75.0% (72 of 96)	0.708	1.1% (6 of 564)	0.0% (0 of 195)	0.142
Metabolic acidosis	42.5% (37 of 87)	35.8% (29 of 81)	0.376	31.3% (225 of 718)	35.7% (75 of 210)	0.231
- Bicarbonate treatment	62.5% (50 of 80)	61.5% (59 of 96)	0.892	48.0% (271 of 564)	55.4% (108 of 195)	0.075
Hypophosphatemia	34.1% (29 of 85)	22.7% (20 of 88)	0.097	3.0% (22 of 729)	1.9% (4 of 207)	0.394
- Phosphate therapy	38.8% (31 of 80)	67.7% (65 of 96)	< 0.001	0.2% (1 of 564)	2.6% (5 of 195)	0.001
Hypocalcemia	67.2% (39 of 58)	59.5% (50 of 84)	0.353	37.6% (160 of 425)	31.4% (64 of 204)	0.129
- Calcium therapy	7.5% (6 of 80)	8.3% (8 of 96)	0.845	24.3% (137 of 564)	3.6% (7 of 195)	< 0.001
Vitamin D therapy	50.0% (40 of 80)	82.3% (79 of 96)	< 0.001	75.4% (425 of 564)	86.2% (168 of 195)	0.002
- Calcitriol	41.3% (33 of 80)	45.8% (44 of 96)	0.550	43.8% (247 of 564)	28.7% (56 of 195)	< 0.001
- Cholecalciferol	13.8% (11 of 80)	66.7% (64 of 96)	< 0.001	28.9% (163 of 564)	79.0% (154 of 195)	< 0.001
Hypertension therapy	25.6% (21 of 82)	28.1% (27 of 96)	0.709	67.1% (432 of 644)	63.8% (127 of 199)	0.390
rhGH therapy	52.1% (49 of 94)	50.0% (50 of 99)	0.771	30.6% (236 of 771)	27.5% (61 of 222)	0.374
Carnitine therapy	47.5% (38 of 80)	58.3% (56 of 96)	0.154	-	-	n.a.
Cysteamine treatment	100.0% (92 of 92)	100.0% (99 of 99)	n.a.	-	-	n.a.
- Immediate release (IR)	97.8% (90 of 92)	34.3% (34 of 99)	< 0.001	-	-	n.a.
- Delayed release (DR)	0.0% (0 of 92)	15.2% (15 of 99)	< 0.001	-	-	n.a.
- Switch from IR to DR	2.2% (2 of 92)	50.5% (50 of 99)	< 0.001	-	-	n.a.

*N* number of conducted measurements

Abbreviations: *INC* infantile nephropathic cystinosis, *CKD* chronic kidney disease, *EPO* erythropoietin, *rhGH* recombinant human growth hormone, *n.a.* not applicable

### Anthropometric characteristics

**Fig. 2:**
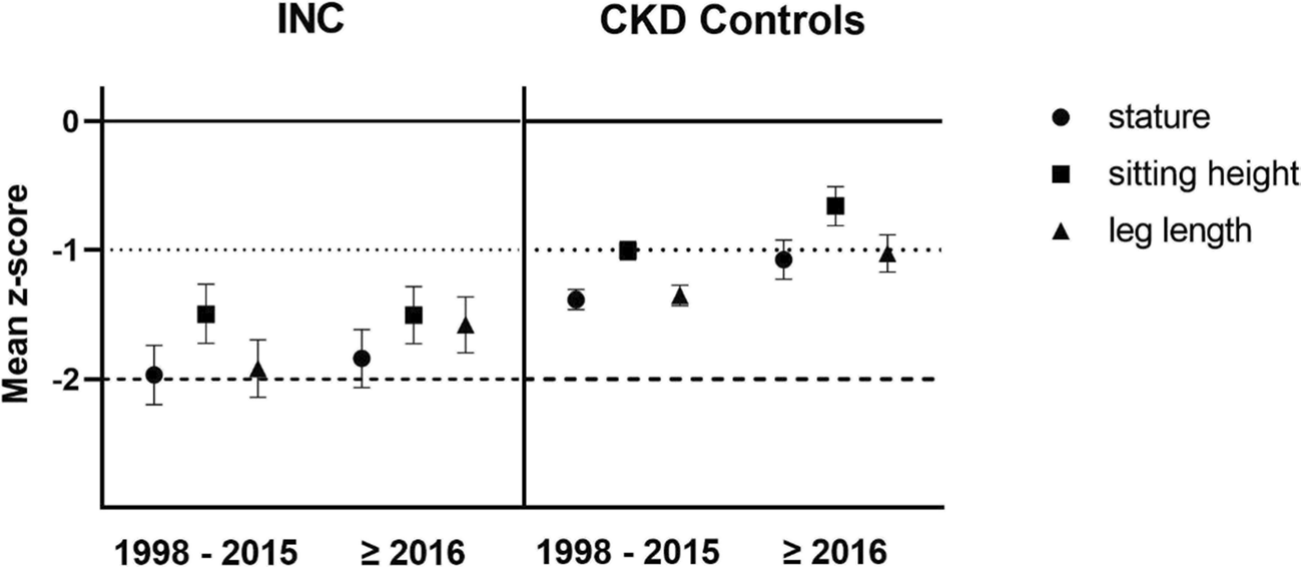
Stature (circle), sitting height (square), and leg length (triangle) in 64 INC and 302 CKD patients, all treated conservatively. Data is presented for the measuring periods 1998–2015 and ≥ 2016 as age- and sex-dependent SDS values (*z*-scores). Error bars represent 95% confidence intervals

Linear growth of INC and CKD patients is presented in Fig. 2 and shows major impairment in all parameters in children with INC over the course of the two measurement clusters. INC and CKD patients presented with similar linear body pattern in the first measuring cluster, with sitting height as the best preserved parameter (INC − 1.69, CKD − 1.03 *z*-score) and overall short stature (INC − 2.22, CKD − 1.44 *z*-score) as a result of markedly impaired leg length (INC − 2.12, CKD − 1.42 *z*-score). In the second cluster, CKD patients presented with synchronized improvement in all three parameters (statistically significant in leg length and stature, both *p* < 0.01).

Contrarily, INC patients exhibited uncoupled growth dynamics in leg length and sitting height, with a significant increase in leg length from − 2.12 to − 1.76 *z*-score (*p* < 0.05) and a decrease in sitting height (− 1.69 to − 1.85 *z*-score), resulting in improved stature (− 2.22 to − 2.09 *z*-score, Fig. 2). Both latter tendencies did not reach levels of statistical significance (*p* > 0.05).

**Fig. 3:**
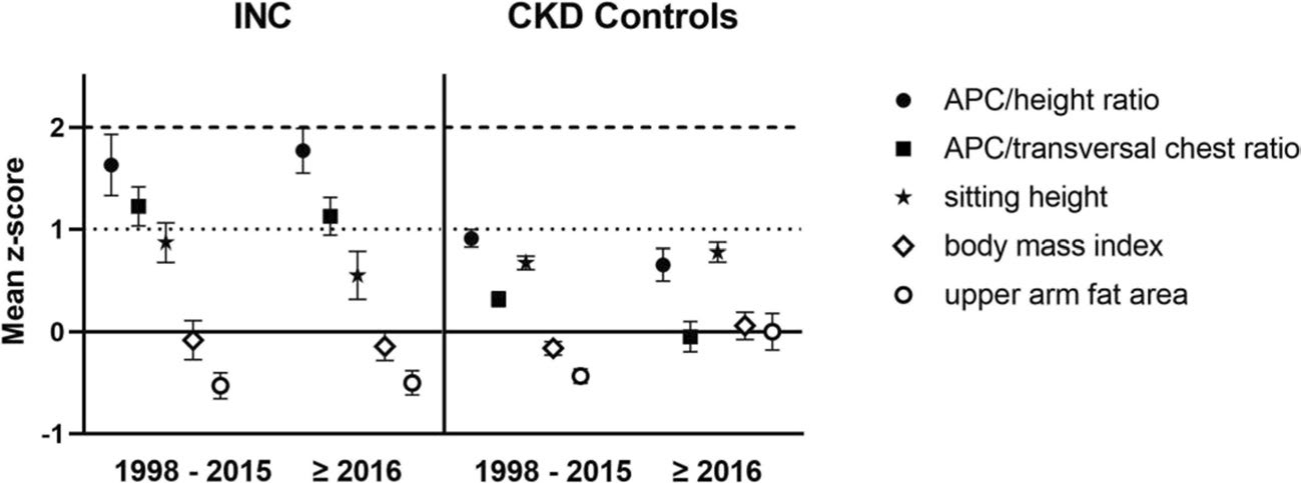
Mean SDS values of anterior–posterior chest/height ratio (APC/height ratio; filled circle), anterior–posterior chest/ transversal ratio (APC/transversal ratio; filled square), sitting height index (star), body mass index (empty diamond), and upper arm fat area (empty circle) in 64 INC and 302 CKD patients, all treated conservatively. Data is presented for the measuring periods 1998–2015 and ≥ 2016 as age- and sex-dependent SDS values (*z*-scores). Error bars represent 95% confidence intervals

Concerning thoracic configurations, which are presented in Fig. 3, both APC/height ratio (2.03 vs. 1.87 *z*-score) and APC/transverse ratio (1.01 vs. 1.57 *z*-score) remained the two most markedly elevated parameters in children with INC over the complete observation period; indicating severe trunk deformation. On the other hand, their peers with CKD presented with more homogeneous *z*-scores of the different parameters.

**Fig. 4:**
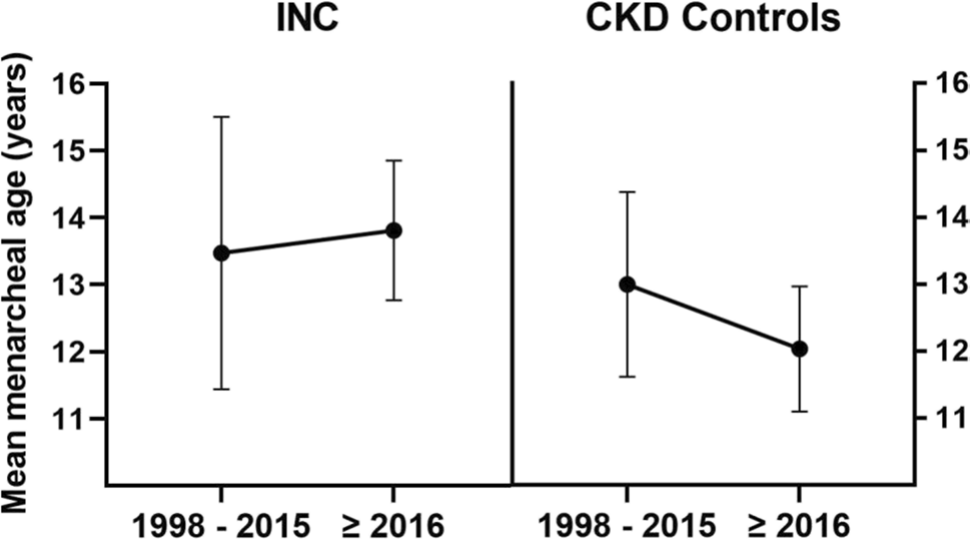
Menarcheal age of 64 INC and 302 CKD patients, all treated conservatively. Data is presented for the measuring periods 1998–2015 and ≥ 2016. Error bars represent 95% confidence intervals.

In patients with INC from 1998 onwards, upper arm fat area (UFA) (− 0.48 to − 0.60 *z*-score) and BMI (0.05 to − 0.37 *z*-score) declined, though only the decline in BMI reached levels of statistical significance. Contrarily, in their peers with CKD, BMI (− 0.16 to 0.03 *z*-score) as well as UFA (− 0.39 to 0.08 *z*-score) significantly increased over the course of the two measurement clusters (Fig. 3). Accordingly, age at menarche in girls with INC increased from a mean age of 13.47 ± 2.20 to 13.81 ± 1.13 years while conversely, in the CKD group, menarcheal age declined from a mean age of 13.00 ± 1.38 to 12.03 ± 0.93 years after 2015 (Fig. 4). Both tendencies did not reach levels of statistical significance (*p* > 0.05).

As thoracic disproportion and impaired body fat storage were the features that most clearly distinguished INC from CKD patients, we analyzed the delta between most elevated markers of thoracic disproportion (APC/height ratio) and most decreased markers of body fat storage (UFA in INC patients and BMI in CKD patients, respectively). We found that high delta values in children with INC persisted over both measurement clusters (∆2.52 to ∆2.47 *z*-score), while in children with CKD, these values markedly declined (∆1.19 to ∆0.88 *z*-score), indicating a synchronization and harmonization of body proportions and composition in this group. The findings of persisting elevated delta values in patients with INC hint at a sustained striking disharmony of thoracic shape and body composition (Fig. 3).

### Associations between leg length, APC/height ratio, UFA, and clinical/biochemical parameters

As an increase in leg length in INC patients over time was the most striking change in morphology, together with consistently reduced upper arm fat area *z*-scores and elevated APC/height ratios, associations between the aforementioned anthropometric parameters and clinical/biochemical parameters were analyzed using linear mixed models (Table 3). The number of significant associations between leg length and clinical parameters declined in both groups (INC and CKD) compared to the respective earlier measurement cluster, but most strikingly, UFA in INC patients was not significantly associated with any of the clinical parameters in either of the measurement clusters. Increased INC and CKD patient age associated significantly with higher leg length. This held true across both measurement clusters. In the earlier cluster, lower APC/height ratio was significantly associated with increasing age in CKD patients. In both CKD clusters, UFA was significantly positively associated with increasing age. eGFR was positively associated with leg length in both CKD measurement clusters, but only in the earlier INC measurement cluster. In CKD patients, improved kidney function was associated with improved UFA in the first cluster. Leg length was significantly positively associated with hemoglobin and phosphate levels in the earlier measurement cluster in patients with INC. CKD patients with high ALP levels exhibited improved leg length in the first cluster while increased bicarbonate was associated with impaired leg length. In the subsequent cluster, sodium was associated with impaired leg length. Regarding thoracic configuration, APC/height ratio in the INC group was significantly positively associated with bicarbonate and phosphate levels, while hemoglobin was significantly negatively associated in the first cluster. In the ensuing cluster, higher sodium levels were associated with decreased APC/height ratio in INC patients. In their peers with CKD, the only observed significant association between APC/height ratio and biochemical parameters was observed with bicarbonate which showed negative associations with APC/height ratio in the second measurement cluster. Corresponding with our findings of persisting reduced body fat stores in children with INC (Fig. 3), none of the observed clinical parameters showed any significant association in INC patients with UFA in both measurement clusters. In CKD patients, higher hemoglobin levels as well as lower sodium levels were associated with increased UFA *z*-scores in the second measurement cluster.

**Table 3:** Associations between clinical (with age) and morphological characteristics (leg length, anterior–posterior chest/height ratio, and upper arm fat area) in INC and CKD patients measured between 1998 and 2015 and after 2015 (linear mixed-effect models applied)

	INC *ß-*values (95% CI)		CKD control *ß*-values (95% CI)	
	1998–2015	≥ 2016	1998–2015	≥ 2016
Leg length				
Intercept	− 8.16 (− 16.40 to 0.08)	− 7.18 (− 14.13 to − 0.22)*	− 0.24 (− 2.82 to 2.34)	2.63 (− 3.16 to 8.42)
Age	0.07 (0.01 to 0.12)*	0.12 (0.04 to 0.21)**	0.08 (0.06 to 0.10)**	0.07 (0.03 to 0.11)**
eGFR (×10)	0.21 (0.07 to 0.35)**	0.07 (− 0.05 to 0.19)	0.07 (0.02 to 0.13)*	0.15 (0.06 to 0.23)**
HCO_3_ (×10)	0.28 (− 0.26 to 0.81)	− 0.16 (− 1.06 to 0.74)	− 0.22 (− 0.43 to 0.01)*	0.33 (− 0.14 to 0.81)
Hemoglobin	0.16 (0.05 to 0.27)**	0.19 (− 0.01 to 0.38)	0.03 (-0.01 to 0.07)	0.04 (− 0.06 to 0.13)
Sodium (×10)	0.10 (− 0.53 to 0.73)	0.11 (− 0.42 to 0.65)	− 0.16 (− 0.34 to 0.02)	− 0.42 (− 0.82 to − 0.01)*
Potassium	0.21 (− 0.13 to 0.55)	0.04 (− 0.35 to 0.43)	− 0.01 (− 0.12 to 0.11)	− 0.07 (− 0.28 to 0.14)
Calcium *z*-score (×10)	0.09 (− 0.25 to 0.44)	0.10 (− 1.04 to 1.25)	0.04 (− 0.12 to 0.20)	− 0.05 (− 0.67 to 0.56)
Phosphate *z*-score	0.23 (0.11 to 0.36)**	− 0.10 (− 0.26 to 0.05)	0.04 (− 0.01 to 0.09)	− 0.04 (− 0.13 to 0.06)
ALP *z*-score (×10)	0.19 (− 0.55 to 0.93)	0.79 (− 0.73 to 2.31)	0.38 (0.04 to 0.72)*	0.11 (− 0.41 to 0.63)
PTH* z*-score (×10)	0.02 (− 0.68 to 0.72)	− 0.06 (− 1.10 to 0.98)	0.13 (− 0.15 to 0.42)	0.49 (− 0.11 to 1.10)
APC/height ratio				
Intercept	1.49 (− 5.52 to 8.49)	15.18 (7.29 to 23.07)**	0.34 (− 3.97 to 4.64)	3.78 (− 5.48 to 13.04)
Age (×10)	− 0.12 (− 0.64 to 0.39)	− 0.91 (− 1.84 to 0.03)	− 0.49 (− 0.78 to − 0.20)**	0.30 (− 0.25 to 0.85)
eGFR (×10)	− 0.03 (− 0.18 to 0.11)	− 0.12 (− 0.26 to 0.01)	− 0.05 (− 0.14 to 0.03)	− 0.06 (− 0.18 to 0.06)
HCO_3_ (×10)	1.19 (0.74 to 1.65)**	0.19 (− 0.81 to 1.19)	0.31 (− 0.05 to 0.67)	− 0.78 (− 1.54 to − 0.02)*
Hemoglobin	− 0.13 (− 0.22 to − 0.03)*	− 0.04 (− 0.25 to 0.18)	− 0.03 (− 0.09 to 0.04)	− 0.06 (− 0.21 to 0.08)
Sodium (×10)	− 0.02 (− 0.55 to 0.52)	− 0.86 (− 1.47 to − 0.26)**	0.02 (− 0.28 to 0.32)	− 0.12 (− 0.76 to 0.53)
Potassium	− 0.09 (− 0.37 to 0.19)	0.13 (− 0.32 to 0.58)	0.09 (− 0.09 to 0.27)	0.24 (− 0.10 to 0.58)
Calcium *z*-score	− 0.01 (− 0.04 to 0.02)	0.04 (− 0.09 to 0.17)	− 0.02 (− 0.05 to 0.01)	− 0.07 (− 0.16 to 0.03)
Phosphate *z*-score(×10)	1.27 (0.21 to 2.33)*	− 0.02 (− 1.75 to 1.72)	0.07 (− 0.76 to 0.89)	− 0.36 (− 1.87 to 1.16)
ALP *z*-score	− 0.01 (− 0.07 to 0.05)	− 0.03 (− 0.20 to 0.14)	− 0.01 (− 0.06 to 0.05)	0.01 (− 0.07 to 0.10)
PTH *z*-score	0.06 (− 0.01 to 0.12)	0.07 (− 0.05 to 0.19)	0.02 (− 0.02 to 0.07)	0.02 (− 0.08 to 0.11)
Upper arm fat area				
Intercept	3.11 (− 10.98 to 17.19)	2.14 (− 5.12 to 9.41)	− 0.92 (− 5.02 to 3.17)	9.24 (− 1.78 to 20.27)
Age (×10)	0.27 (− 0.57 to 1.11)	0.00 (− 0.65 to 0.64)	0.74 (0.47 to 1.02)**	1.49 (0.80 to 2.17)**
eGFR (×10)	0.09 (− 0.11 to 0.29)	0.01 (− 0.09 to 0.11)	0.08 (0.01 to 0.15)*	− 0.02 (− 0.17 to 0.12)
HCO_3_ (×10)	0.42 (− 0.49 to 1.33)	− 0.16 (− 1.01 to 0.68)	− 0.32 (− 0.66 to 0.02)	− 0.52 (− 1.43 to 0.38)
Hemoglobin	− 0.08 (− 0.27 to 0.11)	0.12 (− 0.05 to 0.29)	0.02 (− 0.04 to 0.08)	0.28 (0.11 to 0.46)**
Sodium (×10)	− 0.31 (− 1.39 to 0.77)	− 0.38 (− 0.93 to 0.18)	− 0.04 (− 0.33 to 0.25)	− 0.93 (− 1.70 to − 0.16)*
Potassium	0.03 (− 0.56 to 0.61)	0.29 (− 0.10 to 0.68)	0.09 (− 0.08 to 0.27)	0.04 (− 0.37 to 0.45)
Calcium *z*-score (×10)	0.38 (− 0.21 to 0.98)	− 1.04 (− 2.10 to 0.01)	− 0.01 (− 0.27 to 0.25)	0.31(− 0.84 to 1.46)
Phosphate *z*-score	0.10 (− 0.10 to 0.31)	− 0.08 (− 0.21 to 0.05)	− 0.03 (− 0.11 to 0.05)	0.06 (− 0.12 to 0.24)
ALP *z*-score	0.02 (− 0.10 to 0.14)	− 0.05 (− 0.18 to 0.07)	0.03 (− 0.03 to 0.08)	0.02 (− 0.08 to 0.12)
PTH *z*-score	0.01 (− 0.10 to 0.12)	0.05 (− 0.05 to 0.14)	0.03 (− 0.01 to 0.08)	0.00 (− 0.12 to 0.12)

Data is presented as *ß*-values (95% confidence intervals). ×10 in the first column (e.g. eGFR ×10) signifies extrapolation by factor 10 for improved representation. *p < 0.05; **p < 0.01. Note: Algebraic sign in *ß*-values expresses positive ( +) or negative ( −) association; e.g., higher hemoglobin levels in the INC group measured between 1998 and 2015 are significantly associated with improved leg length growth (*p* < 0.01)

Abbreviations: *INC* infantile nephropathic cystinosis, *CKD* chronic kidney disease, *eGFR* estimated glomerular filtration rate, *ALP* alkaline phosphatase, *PTH* parathyroid hormone, *APC* anterior–posterior chest

## Discussion

Over the past 25 years, both INC patients and CKD patients underwent changes in their morphology and clinical presentation. However, while body morphology in patients with CKD retained homogeneity between individual parameters, children with INC retained disproportion in their thoracic shape, as well as markedly more reduced fat stores, and also exhibited striking changes in leg length over time. While those findings still show INC-specific challenges in growth and clinical development, the significant increase in mean age of conservatively treated INC patients from first to second measurement cluster (*p* < 0.001) illustrates the drastic improvement in prognosis in recent times and shift towards conservative treatment modalities. Furthermore, the mean age in our conservatively treated sample measured after 2015 has exceeded the former age at kidney failure described in historic works [5, 12, 24]; highlighting the dynamic shift in prognosis over past years, making it feasible and relevant to ask questions about the development of affected children into adulthood.

These findings are in accordance with studies showing that the onset of kidney failure can be delayed, and incidence of KRT in the pediatric INC cohort can be reduced by adequate treatment with cysteamine, preferably initiated in the first 5 years of life [13] or even earlier, in the first 2 years [25]. Furthermore, children who started cysteamine therapy before 2 months of age had higher mean eGFR rates at all times than their peers who started therapy later [26]. However, there seems to be no defined age and every month counts. We observed a drastic decrease in age at diagnosis, as well as age at therapy initiation from the earlier to the later measurement cluster. Furthermore, a significant approximation of these two time points occurred. This hints at improved diagnostic procedures and more immediate action in the form of cysteamine therapy commencement, which then likely contributes to the improved renal outcomes observed in patients with INC [13, 26]. This raises questions about the possible impact of early treatment on growth and morphology.

As described in recent publications, INC and CKD patients share a similar pattern of body proportions in the prepubertal period with markedly impaired leg length growth resulting in overall short stature [15, 16]. Now, we have shown that this childhood INC pattern shared with CKD peers was also the apparent morphological pattern in the earlier INC measurement cluster of this analysis, where patients were, incidentally, younger in age. Only in recent years (≥ 2016), was the morphology in INC patients first shown to deviate from this pattern, with a significant increase in leg length, resulting in noticeably increased stature compared to earlier time points (Fig. 2). Interestingly, the number of significant associations between the parameter most distinctive between both patterns, i.e. leg length, and respective clinical parameters markedly declined between time clusters (1998–2015 vs. ≥ 2016) in INC patients (Table 3). This suggests that factors exceeding the current common observational parameters of INC come into play in influencing leg length.

Another striking finding from the analysis of associations between clinical parameters and morphological characteristics was the complete absence of any associations with UFA in INC patients over both clusters. Furthermore, body fat stores and BMI declined even further over the course of the past 25 years, though only BMI did so significantly, despite the fact that all children in the study received dietary counselling to obviate malnutrition caused by an inadequate diet. Possible explanations for diminished body fat stores in the past mostly surrounded gastrointestinal causes such as vomiting and nausea [6], a now known side effect of cysteamine therapy possibly caused by the increased release of gastric acid after ingestion [27], but with the introduction of DR cysteamine, these side effects were reduced [28]. Still, it is important to note that vomiting and nausea were already observed in INC patients before the introduction of cysteamine [6], so the medication side effects may have only contributed to an already existing problem. It seems that the issue of reduced body fat amounts, a characteristic of INC patients already described in the first report dating back to the early twentieth century [3], is linked to underlying causes that remain largely uncertain.

As fat storage is closely linked to the onset of menarche and maturation [29–31], the further decline of UFA to − 0.60 *z*-score in the second cluster can be considered one possible reason for the statistically non-significant delay in onset of menarche observed in our sample (Fig. 4). These findings are in accordance with theories by Frisch and McArthur, specifying a certain percentage of total body fat required for menarche, possibly undercut in girls with INC [32].

Not only did body composition show striking irregularities in children with INC, but body proportions deviated markedly from normal levels, too. Especially, the extremely elevated ratios of thoracic depth to width and height ratios clearly distinguished INC patients from their CKD peers and are a hallmark of disease morphology, as first described by Müller et al. [16]. Furthermore, thoracic diameters have been shown to increase with age, contributing even further to thoracic deformation with a conical chest shape [16]. These morphological irregularities could restrictively impair respiratory mechanics with ensuing pulmonary insufficiency [16, 33], which has been found to affect up to 69% of adult INC patients in a study by Gahl et al. [24]. Thoracic involvement of INC regarding ribcage configuration and respiration is complicated by reports of general myocyte cystine crystal accumulation [34, 35]. These factors contribute further to the increasing demand for interdisciplinary follow-up with increased life expectancy.

Regarding significant developments in clinical parameters, specifically kidney function, our analysis showed a significant decrease in eGFR in both groups over the course of the two measurement clusters while at the same time, mean age increased significantly (Table 1). We therefore adjusted eGFR for age and found that no significant change was noticeable over the past 25 years in either INC (Est. MM 62.90 mL/min per 1.73 m^2^ vs. 59.35 mL/min per 1.73 m^2^) or in CKD patients (Est. MM 38.71 mL/min per 1.73 m^2^ vs. 36.82 mL/min per 1.73 m^2^). The relatively unchanged eGFR is in accordance with studies showing general improvement of kidney function in children with INC as well as CKD and hint at improved clinical care [13, 36].

As kidney function is intrinsically tied to bone metabolism, the persistent elevated ALP levels in INC patients over the past 25 years were striking. ALP is used as a marker for osteoblast activity and bone formation/mineralization; a process typically impaired in children with cystinosis mineral bone disease (CMBD) [37] as well as in patients with CKD mineral bone disease (CKD–MBD) [38]. This, together with our findings regarding reduced growth in the INC group, is in accordance with the known INC feature of hypophosphatemic rickets, which is normally also known to be accompanied by low PTH levels [7]. Our aforementioned results with persistent impaired growth in the second measurement cluster thus hint at a perseverance of this disease feature into recent times, despite modern treatment protocols. This characteristic may also be possibly linked to the persisting impaired thoracic shape, as children with rickets often also present with typical thoracic deformation patterns, e.g., pectus excavatum [39]. Since increased ALP levels have furthermore been shown to be associated with higher all-cause mortality and kidney failure [40] as well as increased risk of cardiovascular events [41], these elevated ALP values do not only hint at unimproved bone metabolism but they may also contribute to the cardiac problems observed in adult INC patients [24].

Our study is limited by the lack of age group–related analysis [15, 16]; however, examination by age group would have exceeded the scope of this study and not have been reflective of the general overview of the past 25 years we intend to give. Our aim is to provide a new approach to analyzing data obtained over 25 years in this prospective, multicenter observational cohort study regarding growth and development in children with INC and CKD. While CKD patients showed general improvement and harmonization in longitudinal parameters and body composition with an unchanged pattern of body morphology, INC patients presented with an isolated increase in leg length and changed body pattern over time. Trunk deformity and reduced body fat amounts have been, and remain, the most characteristic anthropometric features in INC patients over the past 25 years and seemingly unchanged despite modern treatment protocols. Our findings, particularly the lack of significant associations between assessed clinical parameters and body fat amounts, leave room for further investigation into the underlying causes and potential interventions to improve the care and quality of life of INC patients.

### Supplementary Information

Below is the link to the electronic supplementary material.

Graphical abstract (PPTX 183 KB)

## Data Availability

The data supporting the findings of this study are available on request from the corresponding author. Data is not publicly available due to privacy or ethical restrictions.
