# Supplementary material for: Morphological changes and their associations with clinical parameters in children with nephropathic cystinosis and chronic kidney disease prior to kidney replacement therapy over 25 years
Source: Pediatr Nephrol. 2024 Jun 8;39(10):3067–77. doi: 10.1007/s00467-024-06421-6 (PMC11349855; doi:10.1007/s00467-024-06421-6)
Supplement: Supplementary file 1 — Graphical abstract (PPTX 183 KB) [file 467_2024_6421_MOESM1_ESM.pptx]

## Slide 1
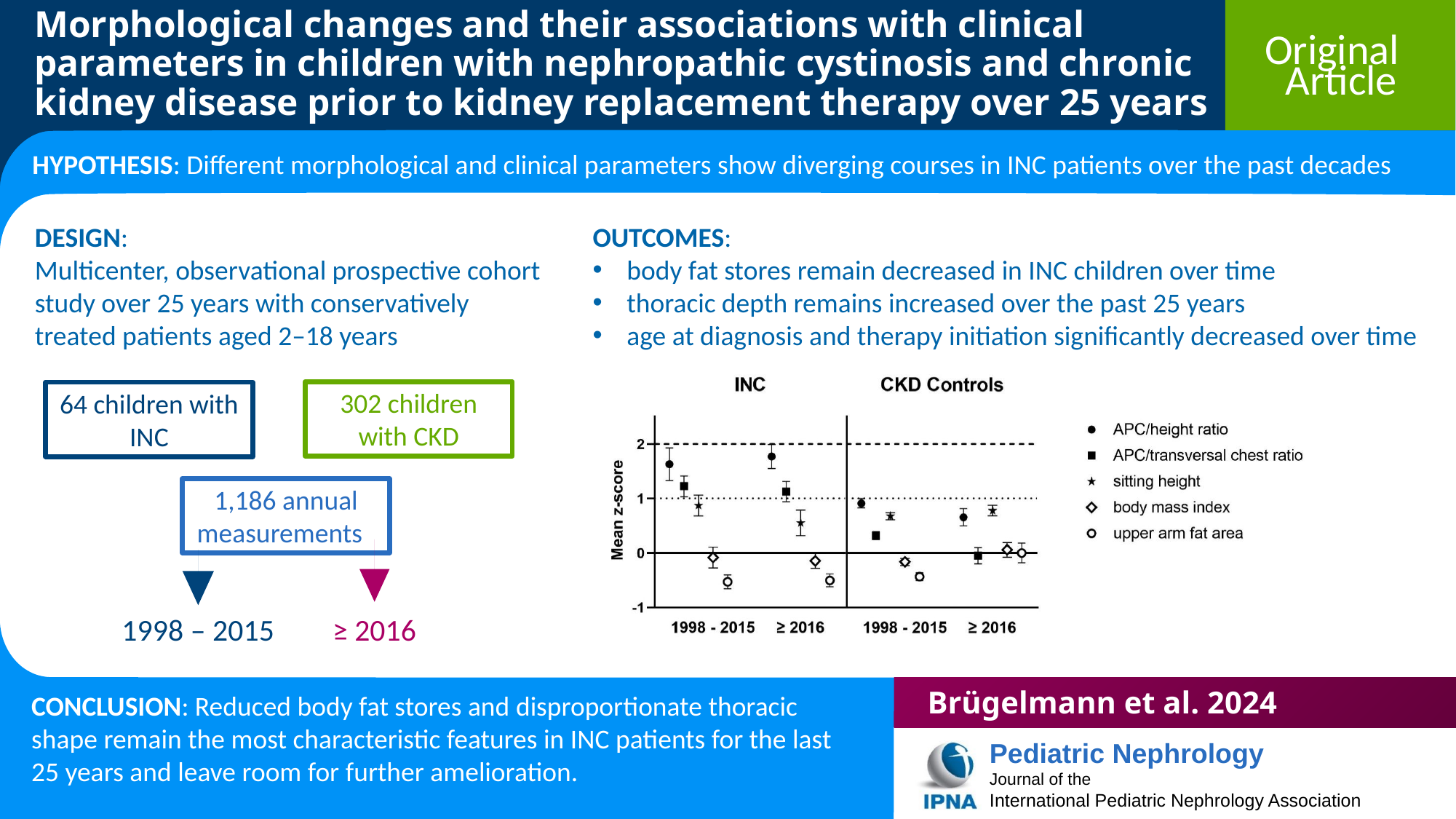

Morphological changes and their associations with clinical parameters in children with nephropathic cystinosis and chronic kidney disease prior to kidney replacement therapy over 25 years
HYPOTHESIS: Different morphological and clinical parameters show diverging courses in INC patients over the past decades
DESIGN:
Multicenter, observational prospective cohort study over 25 years with conservatively treated patients aged 2–18 years
OUTCOMES:
body fat stores remain decreased in INC children over time
thoracic depth remains increased over the past 25 years
age at diagnosis and therapy initiation significantly decreased over time
302 children with CKD
64 children with INC
1,186 annual measurements
1998 – 2015
≥ 2016
Brügelmann et al. 2024
CONCLUSION: Reduced body fat stores and disproportionate thoracic shape remain the most characteristic features in INC patients for the last 25 years and leave room for further amelioration.
